# Routing in the brain

**DOI:** 10.3389/fncom.2014.00044

**Published:** 2014-04-10

**Authors:** Daniel J. Graham

**Affiliations:** Department of Psychology, Hobart and William Smith CollegesGeneva, NY, USA

**Keywords:** routing, connectome, brain network, packet switching, network dynamics, cortex, message switching, BRAIN initiative

As mapping the genome was the great biological challenge a generation ago, so today is mapping brain network dynamics, thanks in part to President Obama's BRAIN initiative (Insel et al., 2013). Factors influencing the emergence of network dynamics, both in the brain and in other networks, can be roughly divided into three classes: those pertaining to node dynamics; those pertaining to topology (connectivity); and those pertaining to routing (how signals are passed across the network). But while single neuron dynamics are reasonably well understood, and while researchers have begun to elucidate key aspects of network topology in brains, very little work has been devoted to possible routing schemes in the brain (Graham and Rockmore, 2011). Indeed, brain networks must possess a systematic routing scheme, but current methods and models often make implicit assumptions about routing—or ignore it altogether.

Routing involves the control of paths that information can take across a network. Given that physical networks have finite limits on links, bandwidth, and memory, the role of routing is to allocate paths such that one or more communication goals are met (e.g., speed, fidelity, fault-tolerance, cost, etc.). Routing is of clear importance for brains: interpreting sensory information, memory access, decision making, and many other core brain functions require that messages can be flexibly sent and received by many nodes at widely separated locations on the network, in response to changing demands.

Now, a paper by Mišić et al. (2014) has simulated communication across a comprehensive macaque cortex anatomical model (CoCoMac: Stephan et al., 2001; Kötter, 2004). Importantly, this study makes explicit assumptions about routing, something that has not previously been done with respect to such detailed connectivity data. The intriguing results of the paper—and the questions regarding routing the paper raises—deserve attention.

Mišić et al. (2014) compare simulated activity on the CoCoMac network with activity on two surrogate network topologies: a generic small world (where any node can communicate with any other over a few “hops”) and a “rich club” (a variety of small world wherein hub nodes have disproportionally dense interconnection and high numbers of shortest paths). Small world structure is recognized as a crucial feature of neural networks, but given evidence of rich club-like topology in cortex (Zamora-López et al., 2010; Van Den Heuvel and Sporns, 2011; Harriger et al., 2012), determining the degree to which cortex shows dynamics characteristic of rich clubs is an important question. However, dynamics depend on topology and routing, so the simulation necessarily involves a routing model.

Mišić et al.'s (2014) surrogate networks were matched to CoCoMac in terms of relevant parameters (nodes, edges, degree, etc.). Sending and receiving nodes, as well as paths between them, were randomly chosen, with new signals introduced according to a Poisson process. Randomized and latticized versions of the networks served as controls.

The results provide evidence that the anatomical network comes closest to the synthetic rich club network in performance, but also shares properties with the small world network (See Figure 1). Mišić et al. (2014) further show that posterior cingulate cortex/precuneus and medial temporal cortex demonstrate congestion characteristic of rich club hub nodes, which matches these regions' proposed roles in integrative functions. The authors also note that “under-congested nodes are areas associated with making eye movements, tracking and acting toward objects in space and fusing visual and proprioceptive information” (Mišić et al., 2014). Thus, there are tantalizing hints of regional or sub-graph variation in network dynamics that correspond to functional demands (albeit in the absence of natural inputs to the system).

**Figure 1:**
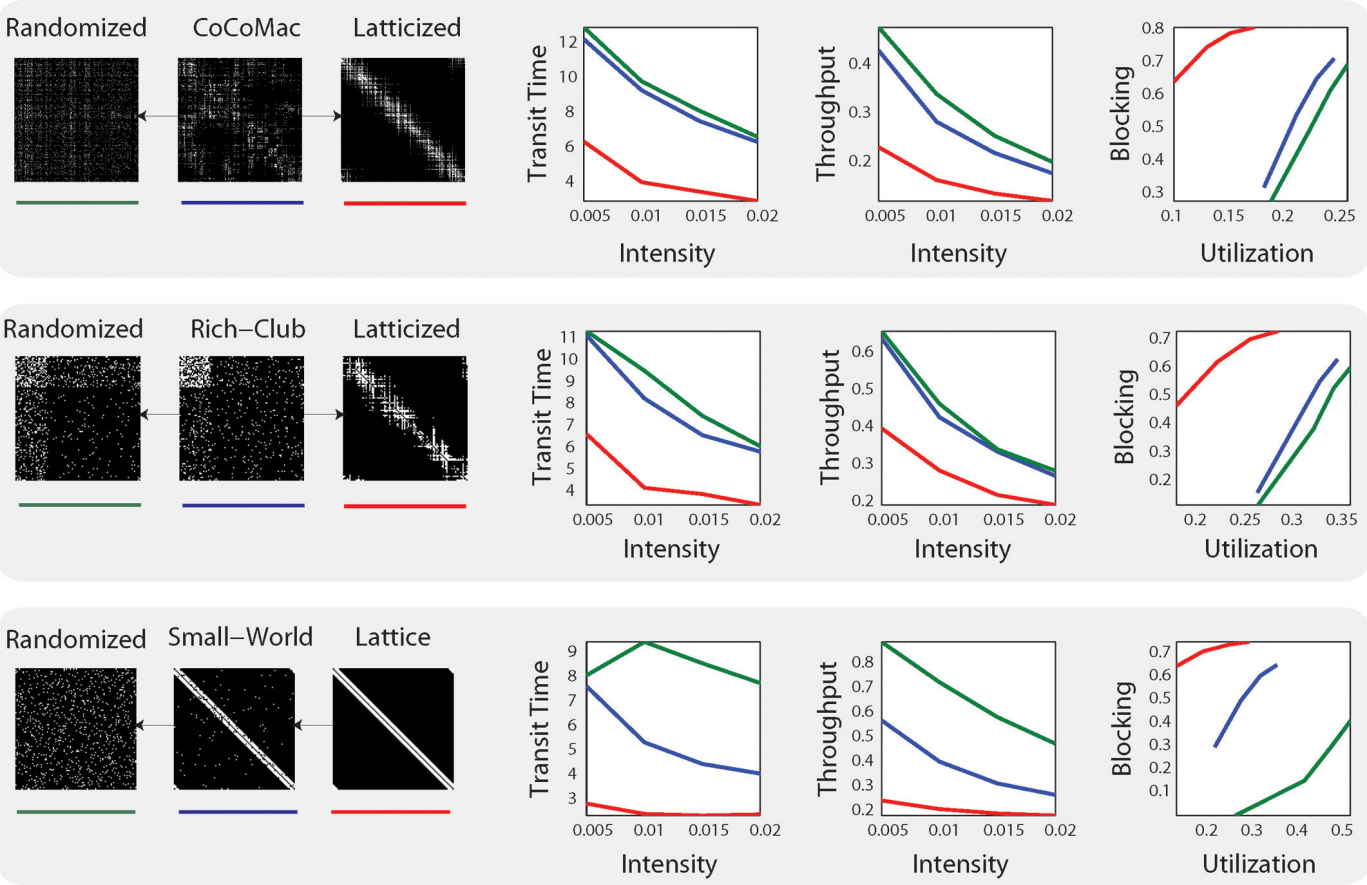
**System-level statistics from Mišić et al. (2014)**. Simulations were carried out on three distinct models: CoCoMac (top), rich-club network (middle) and small-world network (bottom) and their respective randomized (green) and latticized (red) control networks. On the right, mean transit time (the time it takes for a message to travel from source to target), throughput (the total number of deliveries), and utilization (the proportion of time a given brain region is in use) and blocking probability (the probability of losing information) are plotted at four different intensities. Figure from Mišić et al. (2014) Figure 2.

While these findings are compelling, the assumptions made in Mišić et al.'s (2014) routing model are also important. Their model employs message-switched routing, meaning that each signal or “message” is passed along in its entirety from node to node. This scheme is akin to traditional postal systems. But because each node can receive inputs from many other nodes at the same time, messages must “wait their turn” to be passed along. Thus, in message-switched electronic networks, nodes have finite memory buffers to store messages in the queue. There is a high danger of congestion across such networks. Mišić et al.'s (2014) model includes buffers, and the authors provide evidence that buffer size is not a critical parameter: results were not qualitatively different when buffer size was varied over two orders of magnitude. However, the system appears globally inefficient, which may be due to the choice of routing system: transit times and throughput in the simulations declined in tandem with increased load (see Figure 1). This behavior suggests that message switching is not a good match for system demands.

The inefficiency of message-switched architectures is inconsistent with the comparatively rapid and efficient communication typical of real neural networks. Nor is it clear how message switching could be accomplished in real neurons: membrane potential could conceivably store some information about messages in the queue, but it could hardly possess the precision necessary to buffer many “bursty” spike trains, let alone spike timing information.

What alternative routing schemes are plausible in cortex? A circuit-switched network, which is typified by telephone switchboard systems, is another possibility, and is perhaps the default assumption for many modelers and experimentalists. Here, an exclusive path is established between sender and receiver. This system has the advantage of high throughput even under heavy load, and it is this quality that led to its historical dominance in communication systems.

However, such systems are unlikely in brains for four principal reasons:

1. Establishing a path is slow. Sending nodes must first ask the switchboard to provide the connections, and then receiving nodes must send a return signal to acknowledge the connection has been made.
2. The system is inefficient when communication is sparse or intermittent because bandwidth along the path is retained whether or not information is sent along this path.
3. Reorganizing the network is difficult. Because a central operator generally controls the allocation of paths, blockage or destruction of switchboards can lead to network-wide slowdown or blackout.
4. There is neither enough space nor resources in the cranium to support the all-to-all connectivity that would be required to allow exclusive paths between each sender and each receiver.

Thus, it is telling that Mišić et al. (2014) did not countenance the possibility of circuit switching in their simulations. Nevertheless, prominent large-scale cortical models today—despite great power and sophistication—still employ fundamental aspects of circuit switching networks, such as static, centralized routing control (e.g., the DARPA-supported model of Cassidy et al., 2013). The decades-long dominance of the “computer metaphor” in neuroscience may be the inspiration for such models (and they may indeed be appropriate for some local circuits), but it should be clear today that new modes of thinking are necessary to understand brain networks more generally, and cortical networks in particular.

A more promising model for routing in cortex is packet switching, the scheme used on the Internet. Here, messages are chopped into small packets, each labeled with the recipient's address and with what portion of the message that packet contains. The message is reassembled once all constituent packets arrive. Crucially, each packet can take a different route to the destination, allowing the system to dynamically reroute traffic around congested parts of the network. Because activity is distributed in this fashion across a topologically distributed network (and because activity is sparse and bursty), the system functions with high efficiency, and without the need for substantial memory buffers at each node. It is thus a more realistic scheme given the properties of real neurons and neural networks. As described in greater detail by Graham and Rockmore (2011), packet switching has appealing parallels with cortical signaling, for example in

1. Its ability to dynamically reroute traffic, as cortex does following lesion;
2. Its capacity for different “applications” (e.g., email, http, etc.) to run concurrently on the same system, as distinct modalities and signaling systems do in cortex;
3. The inherent hierarchy of the network protocol stack, which mirrors hierarchical organization within and across cortex.

In addition, our evolving understanding of communication in the brain has intriguing parallels with the notion of packet switching. For example, as we begin to unravel the role of glia in neural signaling, there are hints that these cells could act as the routers (Möller et al., 2007).

Of course, the “Internet metaphor” is inexact and it remains to be seen how aspects of this technology could be realized in the brain. For example, addressing would be costly given the relatively small amount of information carried by spikes. However, if most messages travel short distances on the network, addresses may require only a few extra bits. In this case, addresses could be carried by spike timing, while message “content” could be carried by spike rate (Graham and Rockmore, 2011).

Or consider the problem of how a given node can “sense” downstream congestion and reroute signals appropriately. The Internet achieves this in part because a given node (router) receives lists of short paths to popular destinations, which are updated and propagated largely by hub servers (e.g., ISPs). The brain does not appear capable of this. However, the Internet metaphor offers other potential solutions. To detect congestion, the Internet concurrently uses a feedback system involving “acks”: recipient nodes send small feedback messages to the sender “acknowledging” receipt of a tranche of packets. If the sending node does not receive timely acks, it resends lost packets. Analogously in the brain, corticothalamic feedback could conceivably return information about congestion to nodes lower in the hierarchy, which could in turn modify their signaling to compensate if necessary.

Interestingly, Mišić et al. (2014) acknowledge that packet switching is “physiologically plausible” and is a better match to the sparse communication typical of cortex. One therefore hopes these authors and others will investigate packet switching on CoCoMac. In any case, despite the limitations of message-switched architectures, Mišić et al. (2014) provide a useful reference point and inspiration for future studies of routing in the brain.

But there is some degree of irony that, in the absence of large-scale shifts among neuroscientists away from the computer metaphor, computer engineers are themselves beginning to imagine the brain as a packet switched network, rather than an array of transistors. Steve Furber and colleagues (Khan et al., 2008) have built massive processing architectures for neural network simulation that are fundamentally organized around packet-switched routing, which, in addition to granting advantages described above, can be run with low energy costs (Sharp et al., 2012).

Therefore, the time is right for neuroscientists to revisit their assumptions, to take seriously the problem of routing in the brain, and to investigate the possibility that the brain may be more like the Internet than it is like a postal system, a telephone switchboard—or a computer.

## Conflict of interest statement

The author declares that the research was conducted in the absence of any commercial or financial relationships that could be construed as a potential conflict of interest.
